# A Marine Terpenoid, Heteronemin, Induces Both the Apoptosis and Ferroptosis of Hepatocellular Carcinoma Cells and Involves the ROS and MAPK Pathways

**DOI:** 10.1155/2021/7689045

**Published:** 2021-01-04

**Authors:** Wen-Tsan Chang, Yung-Ding Bow, Pei-Jung Fu, Chia-Yang Li, Chang-Yi Wu, Yi-Hua Chang, Yen-Ni Teng, Ruei-Nian Li, Mei-Chin Lu, Yi-Chang Liu, Chien-Chih Chiu

**Affiliations:** ^1^Division of General and Digestive Surgery, Department of Surgery, Kaohsiung Medical University Hospital, Kaohsiung 807, Taiwan; ^2^Department of Surgery, School of Medicine, College of Medicine, Kaohsiung Medical University, Kaohsiung 807, Taiwan; ^3^Digestive Disease Center, Kaohsiung Medical University Hospital, Kaohsiung 807, Taiwan; ^4^Center for Cancer Research, Kaohsiung Medical University Hospital, Kaohsiung Medical University, Kaohsiung 807, Taiwan; ^5^Ph.D. Program in Life Sciences, Kaohsiung Medical University, Kaohsiung 807, Taiwan; ^6^Department of Biotechnology, Kaohsiung Medical University, Kaohsiung 807, Taiwan; ^7^Graduate Institute of Medicine, College of Medicine, Kaohsiung Medical University, Kaohsiung 807, Taiwan; ^8^Department of Biological Sciences, National Sun Yat-Sen University, Kaohsiung 804, Taiwan; ^9^Department of Biological Sciences and Technology, National University of Tainan, Tainan 700, Taiwan; ^10^Department of Biomedical Science and Environment Biology, Kaohsiung Medical University, Kaohsiung 807, Taiwan; ^11^Graduate Institute of Marine Biotechnology, National Dong Hwa University, Pingtung 944, Taiwan; ^12^Division of Hematology-Oncology, Department of Internal Medicine, Kaohsiung Medical University Hospital, Kaohsiung 807, Taiwan; ^13^Department of Internal Medicine, Faculty of Medicine, College of Medicine, Kaohsiung Medical University, Kaohsiung 807, Taiwan; ^14^The Graduate Institute of Medicine, Kaohsiung Medical University, Kaohsiung 807, Taiwan; ^15^Department of Medical Research, Kaohsiung Medical University Hospital, Kaohsiung 807, Taiwan

## Abstract

Hepatocellular carcinoma (HCC) is a leading cause of death, resulting in over 700 thousand deaths annually worldwide. Chemotherapy is the primary therapeutic strategy for patients with late-stage HCC. Heteronemin is a marine natural product isolated from *Hippospongia* sp. that has been found to protect against carcinogenesis in cholangiocarcinoma, prostate cancer, and acute myeloid leukemia. In this study, heteronemin was found to inhibit the proliferation of the HCC cell lines HA22T and HA59T and induce apoptosis via the caspase pathway. Heteronemin treatment also induced the formation of reactive oxygen species (ROS), which are associated with heteronemin-induced cell death, and to trigger ROS removal by mitochondrial SOD2 rather than cytosolic SOD1. The mitogen-activated protein kinase (MAPK) signaling pathway was associated with ROS-induced cell death, and heteronemin downregulated the expression of ERK, a MAPK that is associated with cell proliferation. Inhibitors of JNK and p38, which are MAPKs associated with apoptosis, restored heteronemin-induced cell death. In addition, heteronemin treatment reduced the expression of GPX4, a protein that inhibits ferroptosis, which is a novel form of nonapoptotic programmed cell death. Ferroptosis inhibitor treatment also restored heteronemin-induced cell death. Thus, with appropriate structural modification, heteronemin can act as a potent therapeutic against HCC.

## 1. Introduction

Natural products are the leading source of chemotherapy drugs [1–4]. Over 70% of the Earth's surface is covered by oceans, which have vast biodiversity and are the origin of life [5]. Natural marine products have been found to have bioactivity against cancer progression [6, 7]; for example, makaluvamines, a group of pyrroloiminoquinone alkaloids isolated from marine sponges, have been identified to induce DNA cleavage [8] and protect against skin cancer [9] and lung cancer [10]. Heteronemin is a metabolite found in the sponge *Hippospongia* sp. that exerts potent effects to inhibit carcinogenesis in cholangiocarcinoma [11], prostate cancer [12, 13], and acute myeloid leukemia (AML) [14]. Although the mechanism by which heteronemin inhibits cancer is not entirely clear, heteronemin has been found to regulate the Bcl-mediated apoptotic pathway [12, 15] and autophagy [15]. Topoisomerase II, which is associated with DNA replication [16], has also been found to be inhibited by the heteronemin treatment [12], and topoisomerase II inhibition is the mechanism underlying the effect of many clinical anticancer drugs, such as topotecan and irinotecan, which are topoisomerase I inhibitors [17, 18]. Therefore, heteronemin shows adequate potential as an anticancer agent.

Liver cancer is a leading cause of cancer-associated death around the world, particularly in Asia, and caused over 700 thousand deaths worldwide in 2018 [19, 20]. Approximately 80% of all liver cancer cases are classified as hepatocellular carcinoma (HCC) derived from hepatocytes [19]. Although many therapeutics for HCC, including surgery, organ transplantation, and chemotherapy [21], are available, chemotherapy is the major therapeutic strategy for advanced HCC patients [22]. Targeted therapy is a new approach to chemotherapy that utilizes small molecules or antibodies to target cancer-specific markers and results in cytotoxicity and cell death [23]. Heteronemin was found to target Ras signaling and downregulate NF*κ*B, thus showing potential as a targeted therapeutic agent [14]. A major outcome of chemotherapy is apoptosis, which is the fundamental programmed cell death process [24]. Loss of apoptotic pathways commonly occurs in cancer and results in the survival of tumor cells. Therefore, chemotherapy often targets apoptosis [25]. In recent years, a novel form of programmed cell death called “ferroptosis,” which is iron-dependent cell death that is associated with reactive oxygen species (ROS) and lipid peroxides, has been found to induce cell death and activate inflammation. It underlies the effect of many chemotherapeutic drugs, such as cisplatin [26] and sorafenib [27], which are the first-line treatment for advanced HCC [28].

ROS, including superoxide anions (O_2_∙^–^), hydrogen peroxide (H_2_O_2_), and hydroxyl radicals (∙OH), play a vital role in chemotherapy and mediate several cellular pathways, including apoptosis and ferroptosis. ROS are also associated with the mitogen-activated protein kinase (MAPK) pathway, a conserved regulatory pathway that regulates signal transduction and is involved in several cellular processes, such as proliferation [29], differentiation [30], cell cycle arrest [29], survival [31], and death [32]. Extracellular signal-regulated kinases (ERKs), c-Jun N-terminal kinases (JNKs), and p38 are the major MAPKs, and they respond to stimulation by regulating cell proliferation, apoptosis, ferroptosis, and inflammation [33]. In this study, we demonstrate that the anticancer effect of heteronemin on HCC is associated with ROS-associated MAPK activation and that heteronemin induces HCC death through apoptosis as well as ferroptosis.

## 2. Materials and Methods

### 2.1. Cell Culture

The human HCC lines HA22T/VGH (HA22T, #60168) and HA59T/VGH (HA59T, #60169) were purchased from the Bioresource Collection and Research Center (BCRC; Taiwan) and maintained in Dulbecco's modified Eagle's medium and Ham's F-12 Nutrient Mixture (DMEM/F12, 3 : 2; Gibco; Waltham, MA, USA) supplemented with 8% fetal bovine serum (FBS; Gibco), 2 mM glutamine, and antibiotics at 37°C and 5% CO_2_.

### 2.2. Cell Viability

Cell viability was measured with a trypan blue exclusion assay [34]. Briefly, the treated cells were exposed to 0.2% trypan blue reagent. Viable cells were not stained by the trypan blue dye, and the bright cells were counted as living cells.

### 2.3. Apoptosis Measurement

The HCC cell apoptosis was evaluated by annexin V/7AAD double staining. An apoptosis detection kit (Strong Biotech Corporation, Taipei, Taiwan) was used for annexin V/PI staining according to the manufacturer's instructions. Briefly, the treated cells were harvested, stained with annexin V/7AAD, and analyzed with an LSR II flow cytometer (BD Biosciences, San Jose, CA, USA) and FlowJo 7.6.1 software (Tree Star, Inc., Ashland, OR, USA).

### 2.4. Western Blot Analysis

To evaluate the changes in protein expression, western blotting was performed as follows. Briefly, cells were lysed with lysis buffer and centrifuged at 4°C. The protein concentration was determined by a bicinchoninic acid (BCA) protein assay kit (Pierce, Rockford, IL, USA). Protein lysates (30 *μ*g) were separated by sodium dodecyl sulfate-polyacrylamide gel electrophoresis (SDS-PAGE) and electrotransferred to polyvinylidene difluoride (PVDF) membranes (PALL, Ann Arbor, MI, USA). The membranes were blocked with 5% nonfat milk in TBS-T buffer (TBS buffer containing 0.1% Tween 20) for one hour and incubated with primary antibodies such as Bax (AP1302a, Abgent, San Diego, CA, USA), ERK1/2 (GTX50868, GeneTex, Irvine, CA, USA), SOD1 (Ab13498, Abcam, Cambridge, UK, Eng.), SOD2 (Ab68155, Abcam), GPX4 (Sc-8007, Santa Cruz, Dallas, TX, USA), and *β*-actin (Sc-47778, Santa Cruz) as well as HRP-conjugated secondary antibodies. HRP luminescence was detected with an enhanced chemiluminescence (ECL) detection kit (Amersham Piscataway, NJ, USA).

### 2.5. ROS Detection

Briefly, 2′,7′-dichlorofluorescin diacetate (DCFDA) and dihydroethidium (DHE) were used to detect intracellular H_2_O_2_ and O_2_∙^–^ formation. Treated cells were incubated with 10 *μ*M DCFDA or DHE for 20 minutes. After incubation, the cells were washed with phosphate-buffered saline (PBS) and analyzed by the FlowJo 7.6.1 software (Tree Star, Inc.) and SigmaPlot 11.0 software (Systat Software, San Jose, CA, USA).

### 2.6. Statistical Analysis

Differences between the groups were analyzed by one-way analysis of variance (ANOVA) or Student's *t*-test at least in triplicate. *p* < 0.05 was considered significant.

## 3. Results

### 3.1. Heteronemin Modulates the Proliferation of HCC Cell Lines

**Figure 1:**
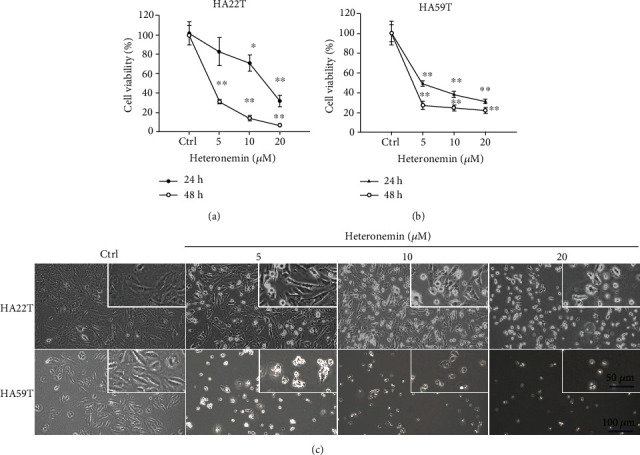
The cytotoxicity of heteronemin against HCC cell lines. The viability of (a) HA22T and (b) HA59T cells was determined 24 and 48 hours after the heteronemin treatment. ^∗∗^*p* < 0.01, ^∗^*p* < 0.05 compared with the control group; all data are presented as the mean ± S.D. of three independent experiments. (c) The morphological changes of HA22T and HA59T cells after 24 hours of heteronemin treatment. Magnification: 100x and 200x.

The cytotoxicity of heteronemin, as a marine drug with potential anticancer effects, was measured in the HCC cell lines HA22T and HA59T. Significant cell death was observed in both HA22T and HA59T cells after the heteronemin treatment, and HA59T cells exhibited higher sensitivity to heteronemin (Figures 1(a) and 1(b)). The IC_50_ values of heteronemin after 24 hours of treatment were 10.4 and 5.25 *μ*M, in HA22T and HA59T cells, respectively. The cell morphological change was also observed after the heteronemin treatment (Figure 1(c)). The results indicated the cytotoxicity of heteronemin in HCC.

### 3.2. Apoptosis Is a Major Regulatory Mechanism Underlying Heteronemin-Associated Programmed Cell Death

**Figure 2:**
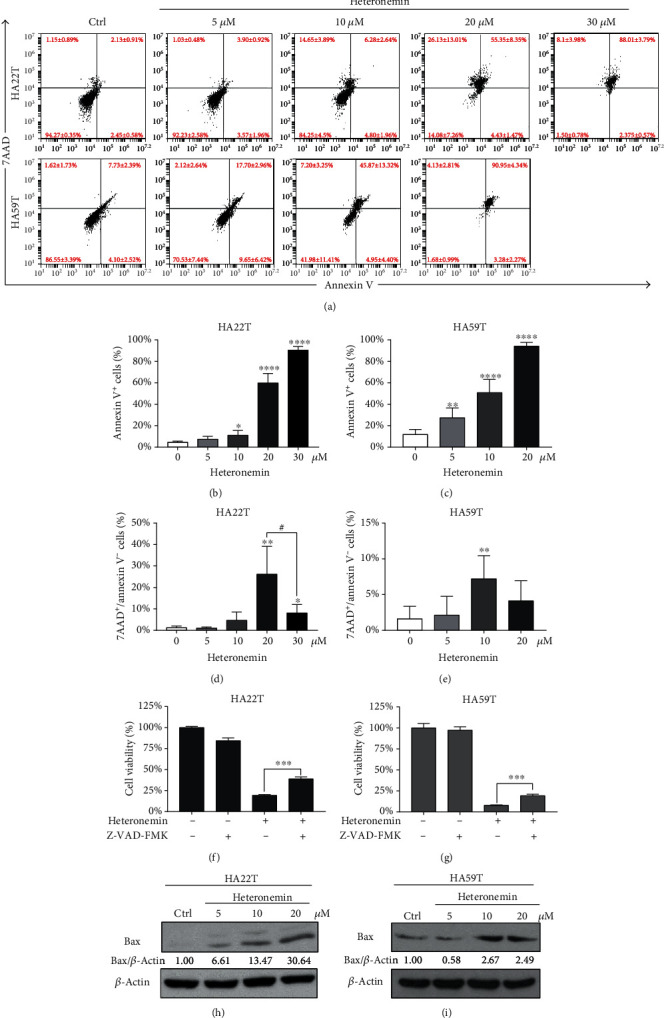
Heteronemin induces cell apoptosis via the caspase cascade. (a) HA22T and HA59T cells were treated with control or 5, 10, 20, or 30 *μ*M heteronemin for 24 hours and stained with annexin V/7AAD to analyze apoptotic cells. (b) and (c) Quantification of apoptotic (annexin V^+^) cells in (a). ^∗^*p* < 0.05, ^∗∗∗∗^*p* < 0.0001 compared with the control. (d) and (e) Quantification of nonapoptotic (annexin V^-/^7AAD^+^) cells in (a). ^∗∗^*p* < 0.01 compared with the control. #*p* < 0.05 compared with 20 *μ*M and 30 *μ*M heteronemin-treated cells. (f) and (g) Cell viability of HA22T and HA59T cells pretreated with 20 *μ*M Z-VAD-FMK, a pan-caspase inhibitor, for 4 hours and treated with 20 *μ*M heteronemin for 24 hours. ^∗∗∗^*p* < 0.001. (h) and (i) Western blot analysis of the Bax expression in heteronemin-treated HA22T and HA59T cells. All data are presented as the mean ± S.D. of three independent experiments.

Apoptosis plays a vital role in the anticancer mechanism of most chemotherapy drugs, such as cisplatin and sorafenib [35, 36]. We stained cells with the apoptosis markers annexin V and 7-amino-actinomycin D (7AAD) to determine whether heteronemin induced apoptosis (Figure 2(a)). Over half of 20 *μ*M heteronemin-treated HA22T and HA59T cells were apoptotic (annexin V^+^) cells, including early-stage apoptotic cells and late-stage apoptotic cells, and HA59T cells were more sensitive than HA22T cells to the effects of heteronemin (Figures 2(b) and 2(c)). In addition, the numbers of annexin V^−^ and 7AAD^+^ nonapoptotic cells were increased in 20 *μ*M heteronemin-treated HA22T cells and 10 *μ*M heteronemin-treated HA59T cells (Figures 2(d) and 2(e)). The caspase family of proteins plays a vital role in apoptosis initiation and progression. To clarify the role of heteronemin-induced apoptosis, we inhibited caspase activity in HA22T and HA59T cells with the pan-caspase inhibitor Z-VAD-FMK. Approximately 20% of growth inhibited by heteronemin was restored by the Z-VAD-FMK treatment (Figures 2(f) and 2(g)). The apoptosis markers cleaved caspase-8, cleaved PARP-1, and Bax were upregulated, and the antiapoptotic protein Bcl2 was downregulated after the heteronemin treatment (Figures 2(h) and 2(i) and Supplementary Figure 1). These data suggested heteronemin showed anticancer potential by activating apoptosis to inhibit cancer growth and induce cell death.

### 3.3. ROS Formation and MAPK/JNK Activation Play a Vital Role in Heteronemin-Mediated Cell Death

**Figure 3:**
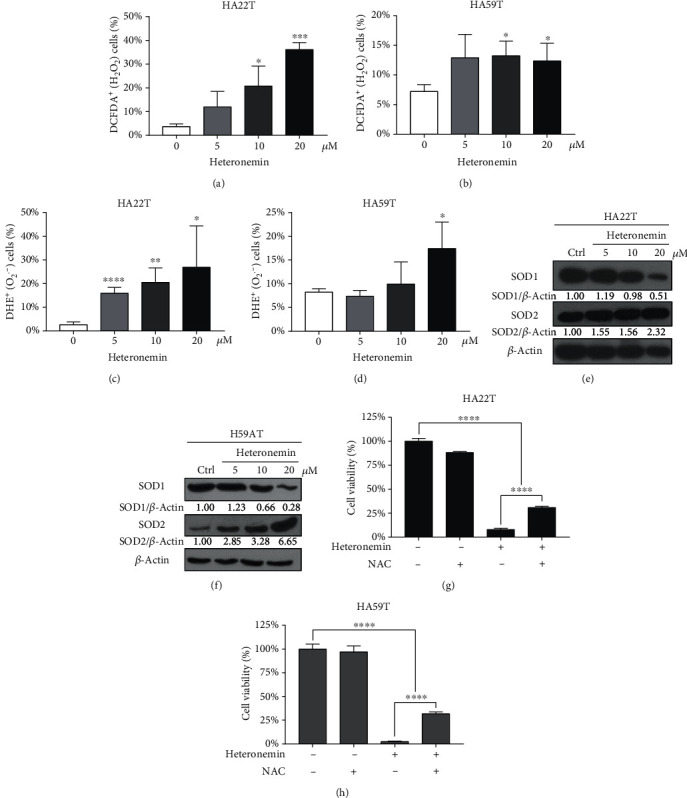
ROS formation is associated with heteronemin-induced cell death. The number of H_2_O_2_-positive cells in (a) HA22T and (b) HA59T was detected with DCFDA and analyzed by flow cytometry. In addition, O_2_∙^–^-positive cells in (c) HA22T and (d) HA59T cells were detected with DHE and analyzed by flow cytometry. Western blot analysis of the SOD1 and SOD2 expression in (e) HA22T and (f) HA59T cells after the heteronemin treatment. (g) HA22T and (h) HA59T cells were treated with NAC (10 mM) for 2 hours before being treated with 20 *μ*M heteronemin, and cell viability was measured after 24 hours. All data are presented as the mean ± S.D. of three independent experiments. ^∗^*p* < 0.05, ^∗∗^*p* < 0.01, ^∗∗∗∗^*p* < 0.0001; all data are presented as the mean ± S.D. of three independent experiments.

ROS are small molecules with high reactivity and play a vital role in many processes that maintain intracellular homeostasis, including autophagy and apoptosis [37, 38]. ROS, such as superoxide anions (O_2_∙^–^), hydroxyl radicals (OH∙), and hydrogen peroxide (H_2_O_2_) [39], are primarily generated during the process of oxidative phosphorylation (OXPHOS), are elevated by many chemotherapeutics, and induce apoptosis [40]. ROS accumulation has been shown to activate the G protein axis, tyrosine kinase receptors, and the p53 pathway and to induce downstream biological pathways depending on the amount of ROS [41]. ROS accumulation also disrupts oxidative balance homeostasis and induces lipid peroxidation, resulting in ferroptosis, which is a novel programmed cell death induced by the disruption of the GSH/GSSH balance [42]. ROS accumulation has been observed in many studies on chemotherapeutic agents, such as 5-fluorouracil, erlotinib, and rituximab, and plays a vital anticancer role [43–45]. To confirm that ROS were formed after heteronemin treatment, 2′,7′-dichlorofluorescein diacetate (DCFDA) and dihydroethidium (DHE) were used to indicate H_2_O_2_ and O_2_∙^–^ formation, respectively. The number of H_2_O_2_ and O_2_∙^–^-positive cells was increased in HA22T and HA59T cells after heteronemin treatment (Figures 3(a)–3(d)). The superoxide dismutase family is associated with the removal of ROS and catalyzing ROS into water and oxygen. Heteronemin treatment downregulated the expression of SOD1 but upregulated the expression of SOD2 (Figures 3(e) and 3(f)). Furthermore, heteronemin-induced cell death was reversed after treatment with the ROS inhibitor N-acetyl-L-cysteine (NAC) (Figures 3(g) and 3(h)).

**Figure 4:**
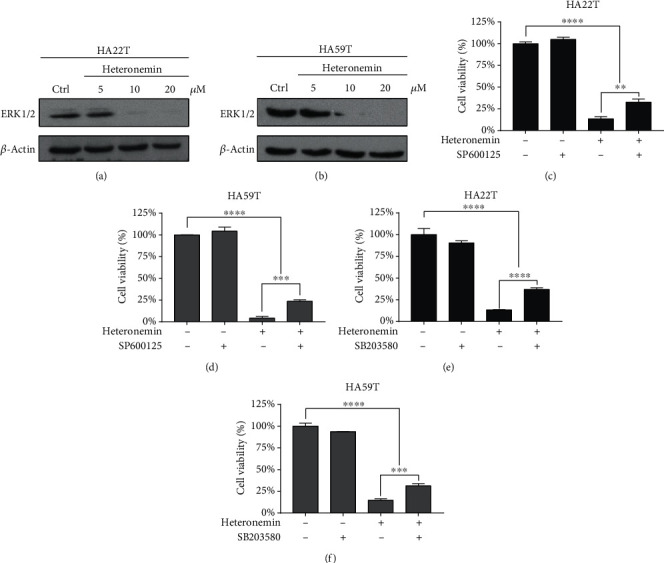
The MAPK signaling pathway regulates the heteronemin-mediated cell death. Western blot analysis of ERK1/2 expression in (a) HA22T and (b) HA59T cells after heteronemin treatment. (c) HA22T and (d) HA59T cells were pretreated with 30 *μ*M SP600125, a JNK inhibitor, for 1 hour before being treated with 20 *μ*M heteronemin, and cell viability was observed. (e) HA22T and (f) HA59T cells were pretreated with 30 *μ*M SB203580, a p38 inhibitor, for 1 hour before being treated with 20 *μ*M heteronemin, and cell viability was analyzed. ^∗∗^*p* < 0.01, ^∗∗∗^*p* < 0.001, ^∗∗∗∗^*p* < 0.0001; all data are presented as the mean ± S.D. of three independent experiments.

Many studies have demonstrated that ROS induce the MAPK signaling pathway and activate caspase-dependent apoptosis. Therefore, we next investigated the role of the MAPK/JNK axis in heteronemin-induced apoptosis. ERK 1/2, classical MAPKs that are activated by growth factors and play critical roles in cell proliferation and tumor progression [46], were downregulated in heteronemin-treated cells (Figures 4(a) and 4(b)). In contrast, the expression of the JNK downstream substrate c-Jun was upregulated, and SP600125, a JNK inhibitor, reversed the heteronemin-induced cell death (Figures 4(c) and 4(d) and Supplementary Figure 1). Additionally, treatment with the p38 inhibitor SB203580 restored the viability of HA22T and HA59T cells after the heteronemin treatment (Figures 4(e) and 4(f)). The results revealed that heteronemin treatment-induced cell death through inducing ROS formation and activating JNK/p38 MAPKs, resulting in cell apoptosis.

### 3.4. Ferroptosis, a Novel Form of Programmed Cell Death, Is Involved in Heteronemin-Induced Cell Death

**Figure 5:**
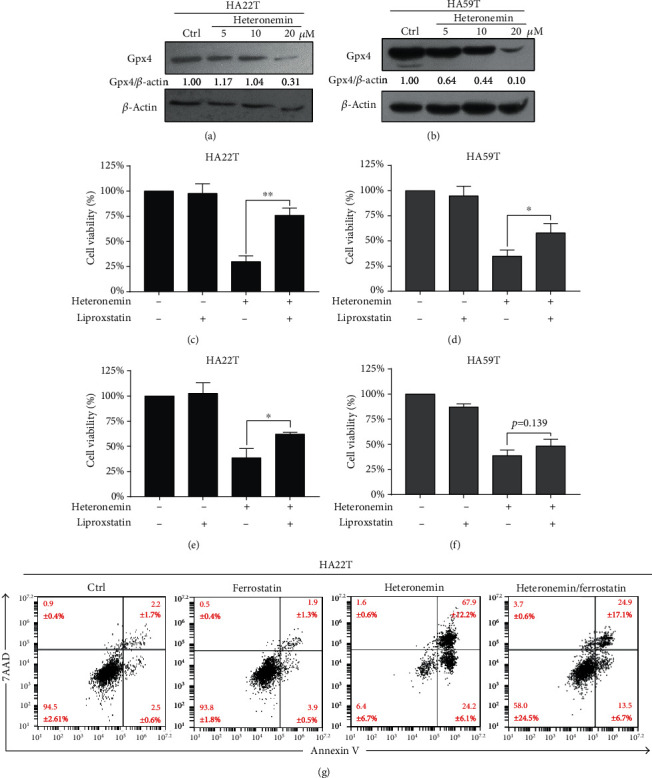
Heteronemin initiates ferroptosis, which is associated with heteronemin-induced cell death. Western blot analysis of ferroptosis markers and the reduction in GPX4 in (a) HA22T and (b) HA59T cells after heteronemin treatment. Liproxstatin and ferrostatin were used to determine the effect of ferroptosis on heteronemin-associated cell death. (c) HA22T and (d) HA59T cells were cotreated with 5 *μ*M liproxstatin and 20 *μ*M heteronemin, and cell viability was measured. (e) HA22T and (f) HA59T cells were cotreated with 15 *μ*M ferrostatin and 20 *μ*M heteronemin treatment, and cell viability was measured. (g) HA22T was cotreated with 15 *μ*M ferrostatin and 20 *μ*M heteronemin, and apoptosis was measured with annexin V/7AAD double staining. ^∗^*p* < 0.05, ^∗∗^*p* < 0.01; all data are presented as the mean ± S.D. of three independent experiments.

Treatment with the caspase inhibitor Z-VAD-FMK or the p38 or JNK inhibitor reduced heteronemin-induced cell death by approximately only 20%. Heteronemin induced cell death not only through apoptosis but also through other forms of programmed cell death. Ferroptosis is a novel form of programmed cell death and is involved in cell death induced by many chemotherapeutics [26, 27]. GPX4 is a vital protein that protects against lipid peroxidation and inhibits ferroptosis initiation, and a reduction in the GPX4 expression is a critical feature of ferroptosis. Cells treated with heteronemin expressed lower levels of GPX4 protein (Figures 5(a) and 5(b)), showing that ferroptosis was involved in heteronemin-induced cell death. Additionally, the ferroptosis inhibitors ferrostatin and liproxstatin reversed heteronemin-induced cell death by approximately 15% (Figures 5(c)–5(f)). Interestingly, treatment with the ferroptosis inhibitor significantly decreased the number of late-stage apoptotic (annexin V^+^/7AAD^+^) cells and increased the proportion of healthy cells (Figure 5(g)). Therefore, like other drugs, heteronemin acts as a potential anticancer drug by inducing cell apoptosis and ferroptosis and may effectively suppress HCC progression.

## 4. Discussion

HCC is a severe disease that causes 700 thousand deaths annually worldwide [19, 20]. In this study, we demonstrated that heteronemin is an effective natural marine product that induces HCC cell proliferation and has potent anticancer potential. Heteronemin was first isolated from *Hyrtios erecta* by Kobayashi et al. in 1994 [47], but research showing that heteronemin induces apoptotic cell death by inhibiting NF-*κ*B activation was not published until 2010 [5]. In recent years, heteronemin has been shown to have anticancer potential in several cancer types by inducing apoptosis, which is usually associated with oxidative stress [11, 12, 48, 49]. Here, we demonstrated that heteronemin has anticancer potential in HCC by inhibiting HA22T and HA59T cell growth and inducing cell apoptosis (Figures 1 and 2).

The ability of heteronemin to induce ROS formation was demonstrated in HCC cell lines (Figures 3(a) and 3(b)). Interestingly, the expression of SOD family proteins, which are essential for ROS removal, was found to be altered. After heteronemin treatment, SOD2 was overexpressed, and SOD1 was downregulated. Similar alterations in expression have been found in C_8_-ceramide-induced apoptosis in lung cancer, and opposing alterations have been observed in breast cancer development [50, 51]. SOD1 is a Zn-Cu-associated dismutase located in the cytoplasm, and SOD2 is a Mn^2+^-associated dismutase located in mitochondria [52]. As shown in Figures 3(e) and 3(f), SOD2 was upregulated, and SOD1 was downregulated in cells in response to heteronemin, showing that mitochondrial oxidative stress is harmful and suggesting that heteronemin may play a role in mitochondrial dysfunction. Consistently, heteronemin was previously found to induce mitochondrial dysfunction and apoptosis in leukemia [49].

The MAPK signaling transduction pathway plays a vital role in various physiological processes and responses to oxidative stress [33]. Three major MAPKs, namely, ERK, JNK, and p38, are involved in this signaling pathway and result in cell proliferation, autophagy, apoptosis, and inflammation. ERK-mediated MAPK signaling has been found to be triggered by stimulation with growth factors (such as epidermal growth factor (EGF) [53]), and the activation of the downstream RAS/RAF/MEK/ERK cascade results in cell proliferation [54]. This cascade is commonly dysregulated in many cancers [55, 56]. ROS-dependent JNK activation has been found to be a robust activator of apoptosis that induces Bcl-Bax signaling and is involved in caspase-dependent apoptosis [57–59]. The ROS/p38/p53 cascade is also a key regulator of cytochrome *c* release, and Bax-initiated caspase activation results in extrinsic and intrinsic (mitochondrial) apoptosis [60–62]. As shown in Figures 4(a) and 4(b), heteronemin effectively reduced the expression level of ERK. On the other hand, treatment with the p38 or JNK inhibitor reversed the cell death caused by heteronemin (Figures 4(c)–4(f)). The results suggested that heteronemin induced ROS formation and initiated apoptosis via the JNK/p38 MAPK signaling pathway.

Ferroptosis is a novel form of programmed cell death associated with oxidative stress, iron accumulation, and lipid peroxidation. Many clinical chemotherapy drugs have been found to not only initiate apoptosis but also induce ferroptosis and protect against cancer growth [63–65]. In addition, immunotherapy has also been found to regulate ferroptosis by enhancing the accumulation of lipid peroxides and regulating the expression of SLC3A2 and SLC7A11, the subunits of the chloride-dependent cystine-glutamate (xCT) antiporter system, which regulates redox homeostasis and oxidative stress [66] to inhibit lipid peroxidation and ferroptosis [67]. GPX4 is a phospholipid-hydroperoxide glutathione peroxidase that protects against lipid peroxidation and ferroptosis [68]. GPX4 is commonly inactivated during ferroptosis [69]. Heteronemin treatment downregulated GPX4, and the ferroptosis inhibitors liproxstatin and ferrostatin significantly reversed heteronemin-induced cell death (Figures 5(a)–5(f)). Interestingly, treatment with the ferroptosis inhibitors liproxstatin and ferrostatin reduced the level of late-stage apoptotic cell death (Figure 5(g)); previous research has shown that annexin V/PI-positive cells may be late-stage apoptotic cells, necroptotic cells, or ferroptotic cells [70–72]. The MAPK signaling pathway has also been found to be involved in ferroptosis initiation. In AML cells, the inhibition of MAPKs, especially p38 and JNK, but not ERK, results in AML insensitivity to erastin [73]. In addition, in 2018, Poursaitidis et al. [74] showed that inhibiting MAPK signaling protects lung cancer cells against ferroptosis. Consistently, MAPKs also play a vital role in heteronemin-induced ferroptosis.

Finally, we performed an animal experiment to validate the anticancer potential of heteronemin *in vivo* (data not shown). We treated mice with three different doses of heteronemin (1 mg/kg, 5 mg/kg, and 10 mg/kg), and tumor volume was significantly reduced after treatment with 1 mg/kg heteronemin; however, due to the cytotoxicity of heteronemin, the 5 mg/kg and 10 mg/kg doses of heteronemin were lethal, and even the mice treated with 1 mg/kg heteronemin died after two weeks of treatment. The results indicated that heteronemin is cytotoxic to HCC cells but also has severe side effects in mice. Thus, it is critical to determine the side effects of heteronemin. In addition, it is crucial to further investigate the cytotoxicity of heteronemin in healthy cells. In this experiment, heteronemin was administered via intraperitoneal injection, which caused the drug to spread to all organs of the mice. Hepatic arterial infusion chemotherapy (HAIC), which directly delivers drugs to tumors and minimizes systemic toxicity, is a feasible strategy for administering heteronemin [75].

## 5. Conclusions

**Figure 6:**
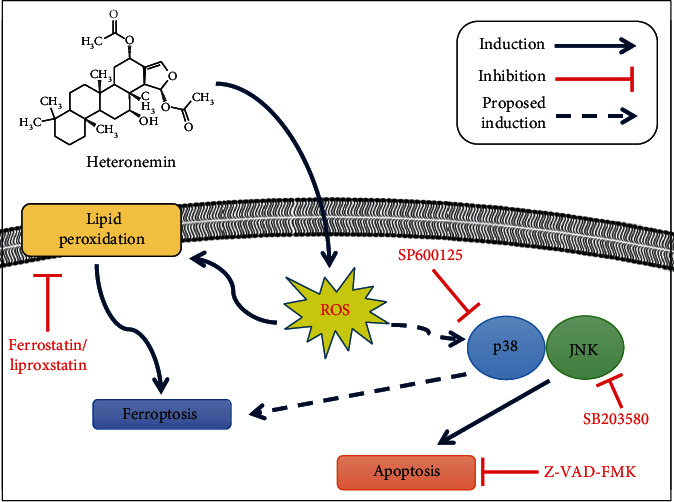
The potential anticancer mechanism of heteronemin. Heteronemin was found to induce ROS formation, resulting in p38/JNK activation and caspase-associated apoptosis and ferroptosis and leading to cancer cell death.

In conclusion, heteronemin is an effective agent against HCC that induces HCC cell apoptosis and ferroptosis by inducing intracellular ROS formation and the p38/JNK MAPK signaling pathway, revealing the potent MAPK-mediated crosstalk mechanism between apoptosis and ferroptosis (Figure 6).

## Data Availability

The data used to support the findings of this study are available from the corresponding author upon request.

## Supplementary Materials

Supplementary Materials Supplementary Figure 1: The expression of apoptotic- and MAPK-associated proteins. HA22T cells were treated with indicated concentrations of heteronemin for 14 hours respectively, and the changes of protein expression was determined by Western blot assay.

## References

[1] von Pawel J., Schiller J. H., Shepherd F. A. (1999). Topotecan versus cyclophosphamide, doxorubicin, and vincristine for the treatment of recurrent small-cell lung cancer. *Journal of Clinical Oncology*.

[2] Miyamoto M., Takano M., Kuwahara M. (2018). Efficacy of combination chemotherapy using irinotecan and nedaplatin for patients with recurrent and refractory endometrial carcinomas: preliminary analysis and literature review. *Cancer Chemotherapy and Pharmacology*.

[3] Demetri G. D., von Mehren M., Jones R. L. (2016). Efficacy and safety of trabectedin or dacarbazine for metastatic liposarcoma or leiomyosarcoma after failure of conventional chemotherapy: results of a phase III randomized multicenter clinical trial. *Journal of Clinical Oncology*.

[4] Kashiwagi S., Asano Y., Goto W. (2018). Mesenchymal-epithelial transition and tumor vascular remodeling in eribulin chemotherapy for breast cancer. *Anticancer Research*.

[5] Schumacher M., Cerella C., Eifes S. (2010). Heteronemin, a spongean sesterterpene, inhibits TNF alpha-induced NF-kappa B activation through proteasome inhibition and induces apoptotic cell death. *Biochemical Pharmacology*.

[6] Khalifa S. A. M., Elias N., Farag M. A. (2019). Marine natural products: a source of novel anticancer drugs. *Marine Drugs*.

[7] Wali A. F., Majid S., Rasool S. (2019). Natural products against cancer: review on phytochemicals from marine sources in preventing cancer. *Saudi Pharmaceutical Journal*.

[8] Matsumoto S. S., Haughey H. M., Schmehl D. M. (1999). Makaluvamines vary in ability to induce dose-dependent DNA cleavage via topoisomerase II interaction. *Anti-Cancer Drugs*.

[9] Cowan J., Shadab M., Nadkarni D. H., Kc K., Velu S. E., Yusuf N. (2019). A novel marine natural product derived pyrroloiminoquinone with potent activity against skin cancer cells. *Marine Drugs*.

[10] Nadkarni D. H., Wang F., Wang W. (2009). Synthesis and in vitro anti-lung cancer activity of novel 1, 3, 4, 8-tetrahydropyrrolo [4, 3, 2-de]quinolin-8(1H)-one alkaloid analogs. *Medicinal Chemistry*.

[11] Lin H.-Y., Tey S. L., Ho Y. (2018). Heteronemin induces anti-proliferation in cholangiocarcinoma cells via inhibiting TGF-*β* pathway. *Marine Drugs*.

[12] Lee M. G., Liu Y. C., Lee Y. L. (2018). Heteronemin, a marine sesterterpenoid-type metabolite, induces apoptosis in prostate LNcap cells via oxidative and ER stress combined with the inhibition of topoisomerase II and Hsp90. *Marine Drugs*.

[13] Wu J. C., Wang C. T., Hung H. C. (2016). Heteronemin is a novel c-met/STAT3 inhibitor against advanced prostate cancer cells. *Prostate*.

[14] Saikia M., Retnakumari A. P., Anwar S. (2018). Heteronemin, a marine natural product, sensitizes acute myeloid leukemia cells towards cytarabine chemotherapy by regulating farnesylation of Ras. *Oncotarget*.

[15] Wu S. Y., Sung P. J., Chang Y. L., Pan S. L., Teng C. M. (2015). Heteronemin, a spongean sesterterpene, induces cell apoptosis and autophagy in human renal carcinoma cells. *BioMed Research International*.

[16] Lucas I., Germe T., Chevrier-Miller M., Hyrien O. (2001). Topoisomerase II can unlink replicating DNA by precatenane removal. *The EMBO Journal*.

[17] Arun B., Frenkel E. P. (2001). Topoisomerase I inhibition with topotecan: pharmacologic and clinical issues. *Expert Opinion on Pharmacotherapy*.

[18] Gilbert D. C., Chalmers A. J., El-Khamisy S. F. (2012). Topoisomerase I inhibition in colorectal cancer: biomarkers and therapeutic targets. *British Journal of Cancer*.

[19] Bray F., Ferlay J., Soerjomataram I., Siegel R. L., Torre L. A., Jemal A. (2018). Global cancer statistics 2018: GLOBOCAN estimates of incidence and mortality worldwide for 36 cancers in 185 countries. *CA: A Cancer Journal for Clinicians*.

[20] Rawla P., Sunkara T., Muralidharan P., Raj J. P. (2018). Update in global trends and aetiology of hepatocellular carcinoma. *Contemporary Oncology*.

[21] Forner A., Reig M., Bruix J. (2018). Hepatocellular carcinoma. *The Lancet*.

[22] Medavaram S., Zhang Y. (2018). Emerging therapies in advanced hepatocellular carcinoma. *Experimental Hematology and Oncology*.

[23] Perez-Herrero E., Fernandez-Medarde A. (2015). Advanced targeted therapies in cancer: drug nanocarriers, the future of chemotherapy. *European Journal of Pharmaceutics and Biopharmaceutics*.

[24] Makin G., Hickman J. A. (2000). Apoptosis and cancer chemotherapy. *Cell and Tissue Research*.

[25] Pfeffer C. M., Singh A. T. K. (2018). Apoptosis: a target for anticancer therapy. *International Journal of Molecular Sciences*.

[26] Guo J., Xu B., Han Q. (2018). Ferroptosis: a novel anti-tumor action for cisplatin. *Cancer Research and Treatment*.

[27] Dixon S. J., Patel D. N., Welsch M. (2014). Pharmacological inhibition of cystine-glutamate exchange induces endoplasmic reticulum stress and ferroptosis. *eLife*.

[28] Ikeda M., Morizane C., Ueno M., Okusaka T., Ishii H., Furuse J. (2018). Chemotherapy for hepatocellular carcinoma: current status and future perspectives. *Japanese Journal of Clinical Oncology*.

[29] Sarnat H. B., Morrissy R. T. (1981). Idiopathic torticollis: sternocleidomastoid myopathy and accessory neuropathy. *Muscle & Nerve*.

[30] Rodríguez-Carballo E., Gámez B., Ventura F. (2016). p38 MAPK signaling in osteoblast differentiation. *Frontiers in Cell and Developmental Biology*.

[31] Mukherjee R., McGuinness D. H., McCall P. (2011). Upregulation of MAPK pathway is associated with survival in castrate-resistant prostate cancer. *British Journal of Cancer*.

[32] Vilela B., Pagès M., Lumbreras V. (2010). Regulation of MAPK signaling and cell death by MAPK phosphatase MKP2. *Plant Signaling & Behavior*.

[33] Son Y., Kim S., Chung H. T., Pae H. O. (2013). Reactive oxygen species in the activation of MAP kinases. *Methods in Enzymology*.

[34] Strober W. (2001). Trypan blue exclusion test of cell viability. *Current Protocols in Immunology*.

[35] Zeng C. W., Zhang X. J., Lin K. Y. (2012). Camptothecin induces apoptosis in cancer cells via microRNA-125b-mediated mitochondrial pathways. *Molecular Pharmacology*.

[36] Fernando J., Sancho P., Fernandez-Rodriguez C. M. (2012). Sorafenib sensitizes hepatocellular carcinoma cells to physiological apoptotic stimuli. *Journal of Cellular Physiology*.

[37] Filomeni G., De Zio D., Cecconi F. (2015). Oxidative stress and autophagy: the clash between damage and metabolic needs. *Cell Death and Differentiation*.

[38] Diwanji N., Bergmann A. (2018). An unexpected friend - ROS in apoptosis-induced compensatory proliferation: implications for regeneration and cancer. *Seminars in Cell & Developmental Biology*.

[39] Moloney J. N., Cotter T. G. (2018). ROS signalling in the biology of cancer. *Seminars in Cell & Developmental Biology*.

[40] Yang H., Villani R. M., Wang H. (2018). The role of cellular reactive oxygen species in cancer chemotherapy. *Journal of Experimental & Clinical Cancer Research*.

[41] Perillo B., Di Donato M., Pezone A. (2020). ROS in cancer therapy: the bright side of the moon. *Experimental & Molecular Medicine*.

[42] Li J., Cao F., Yin H. L. (2020). Ferroptosis: past, present and future. *Cell Death & Disease*.

[43] Alas S., Ng C. P., Bonavida B. (2002). Rituximab modifies the cisplatin-mitochondrial signaling pathway, resulting in apoptosis in cisplatin-resistant non-Hodgkin's lymphoma. *Clinical Cancer Research*.

[44] Hwang P. M., Bunz F., Yu J. (2001). Ferredoxin reductase affects p53-dependent, 5-fluorouracil-induced apoptosis in colorectal cancer cells. *Nature Medicine*.

[45] Shan F., Shao Z., Jiang S., Cheng Z. (2016). Erlotinib induces the human non-small-cell lung cancer cells apoptosis via activating ROS-dependent JNK pathways. *Cancer Medicine*.

[46] Roskoski R. (2012). ERK1/2 MAP kinases: structure, function, and regulation. *Pharmacological Research*.

[47] Kobayashi M., Okamoto T., Hayashi K., Yokoyama N., Sasaki T., Kitagawa I. (1994). Marine natural products. XXXII. Absolute configurations of C-4 of the manoalide family, biologically active sesterterpenes from the marine sponge Hyrtios erecta. *Chemical & Pharmaceutical Bulletin*.

[48] Cheng M. H., Huang H. L., Lin Y. Y. (2019). BA6 induces apoptosis via stimulation of reactive oxygen species and inhibition of oxidative phosphorylation in human lung cancer cells. *Oxidative Medicine and Cellular Longevity*.

[49] Chen Y. C., Lu M. C., El-Shazly M. (2018). Breaking down leukemia walls: heteronemin, a sesterterpene derivative, induces apoptosis in leukemia Molt4 cells through oxidative stress, mitochondrial dysfunction and induction of talin expression. *Marine Drugs*.

[50] Chang Y., Fong Y., Tsai E. M. (2018). Exogenous C8-Ceramide induces apoptosis by overproduction of ROS and the switch of superoxide dismutases SOD1 to SOD2 in human lung cancer cells. *International Journal of Molecular Sciences*.

[51] Papa L., Hahn M., Marsh E. L., Evans B. S., Germain D. (2014). SOD2 to SOD1 switch in breast cancer. *The Journal of Biological Chemistry*.

[52] Zelko I. N., Mariani T. J., Folz R. J. (2002). Superoxide dismutase multigene family: a comparison of the CuZn-SOD (SOD1), Mn-SOD (SOD2), and EC-SOD (SOD3) gene structures, evolution, and expression. *Free Radical Biology & Medicine*.

[53] Huang L., Fu L. (2015). Mechanisms of resistance to EGFR tyrosine kinase inhibitors. *Acta Pharmaceutica Sinica B*.

[54] Liu F., Yang X., Geng M., Huang M. (2018). Targeting ERK, an Achilles' heel of the MAPK pathway, in cancer therapy. *Acta Pharmaceutica Sinica B*.

[55] Roberts P. J., Der C. J. (2007). Targeting the Raf-MEK-ERK mitogen-activated protein kinase cascade for the treatment of cancer. *Oncogene*.

[56] Hobbs G. A., Der C. J., Rossman K. L. (2016). RAS isoforms and mutations in cancer at a glance. *Journal of Cell Science*.

[57] Zhang D., Zhou Q., Huang D. (2019). ROS/JNK/c-Jun axis is involved in oridonin-induced caspase-dependent apoptosis in human colorectal cancer cells. *Biochemical and Biophysical Research Communications*.

[58] Lu Z., Miao Y., Muhammad I. (2017). Colistin-induced autophagy and apoptosis involves the JNK-Bcl2-Bax signaling pathway and JNK-p53-ROS positive feedback loop in PC-12 cells. *Chemico-Biological Interactions*.

[59] Shi Y., Nikulenkov F., Zawacka-Pankau J. (2014). ROS-dependent activation of JNK converts p53 into an efficient inhibitor of oncogenes leading to robust apoptosis. *Cell Death and Differentiation*.

[60] Liu J., Wu N., Ma L. N. (2014). p38 MAPK signaling mediates mitochondrial apoptosis in cancer cells induced by oleanolic acid. *Asian Pacific Journal of Cancer Prevention*.

[61] Liu B., Yuan B., Zhang L., Mu W., Wang C. (2015). ROS/p38/p53/Puma signaling pathway is involved in emodin-induced apoptosis of human colorectal cancer cells. *International Journal of Clinical and Experimental Medicine*.

[62] Park G. B., Choi Y., Kim Y. S., Lee H. K., Kim D., Hur D. Y. (2014). ROS-mediated JNK/p38-MAPK activation regulates Bax translocation in Sorafenib-induced apoptosis of EBV-transformed B cells. *International Journal of Oncology*.

[63] Lachaier E., Louandre C., Godin C. (2014). Sorafenib induces ferroptosis in human cancer cell lines originating from different solid tumors. *Anticancer Research*.

[64] Yang R., Li Y., Wang X. (2019). Doxorubicin loaded ferritin nanoparticles for ferroptosis enhanced targeted killing of cancer cells. *RSC Advances*.

[65] Codenotti S., Poli M., Asperti M., Zizioli D., Marampon F., Fanzani A. (2018). Cell growth potential drives ferroptosis susceptibility in rhabdomyosarcoma and myoblast cell lines. *Journal of Cancer Research and Clinical Oncology*.

[66] Koppula P., Zhang Y., Zhuang L., Gan B. (2018). Amino acid transporter SLC7A11/xCT at the crossroads of regulating redox homeostasis and nutrient dependency of cancer. *Cancer Communications*.

[67] Wang W., Green M., Choi J. E. (2019). CD8(+) T cells regulate tumour ferroptosis during cancer immunotherapy. *Nature*.

[68] Brigelius-Flohe R., Maiorino M. (2013). Glutathione peroxidases. *Biochimica et Biophysica Acta (BBA) - General Subjects*.

[69] Dixon S. J., Winter G. E., Musavi L. S. (2015). Human haploid cell genetics reveals roles for lipid metabolism genes in nonapoptotic cell death. *ACS Chemical Biology*.

[70] Sui X., Zhang R., Liu S. (2018). RSL3 drives ferroptosis through GPX4 inactivation and ROS production in colorectal cancer. *Frontiers in Pharmacology*.

[71] Sun Y., Zheng Y., Wang C., Liu Y. (2018). Glutathione depletion induces ferroptosis, autophagy, and premature cell senescence in retinal pigment epithelial cells. *Cell Death and Disease*.

[72] Pietkiewicz S., Schmidt J. H., Lavrik I. N. (2015). Quantification of apoptosis and necroptosis at the single cell level by a combination of imaging flow cytometry with classical annexin V/propidium iodide staining. *Journal of Immunological Methods*.

[73] Yu Y., Xie Y., Cao L. (2015). The ferroptosis inducer erastin enhances sensitivity of acute myeloid leukemia cells to chemotherapeutic agents. *Molecular and Cellular Oncology*.

[74] Poursaitidis I., Wang X., Crighton T. (2017). Oncogene-selective sensitivity to synchronous cell death following modulation of the amino acid nutrient cystine. *Cell Reports*.

[75] Song M. J. (2015). Hepatic artery infusion chemotherapy for advanced hepatocellular carcinoma. *World Journal of Gastroenterology*.

