# Supplementary figures and images for: A Marine Terpenoid, Heteronemin, Induces Both the Apoptosis and Ferroptosis of Hepatocellular Carcinoma Cells and Involves the ROS and MAPK Pathways
Source: Oxid Med Cell Longev. 2021 Jan 4;2021:7689045. doi: 10.1155/2021/7689045 (PMC7803406; doi:10.1155/2021/7689045)

## Slide 1
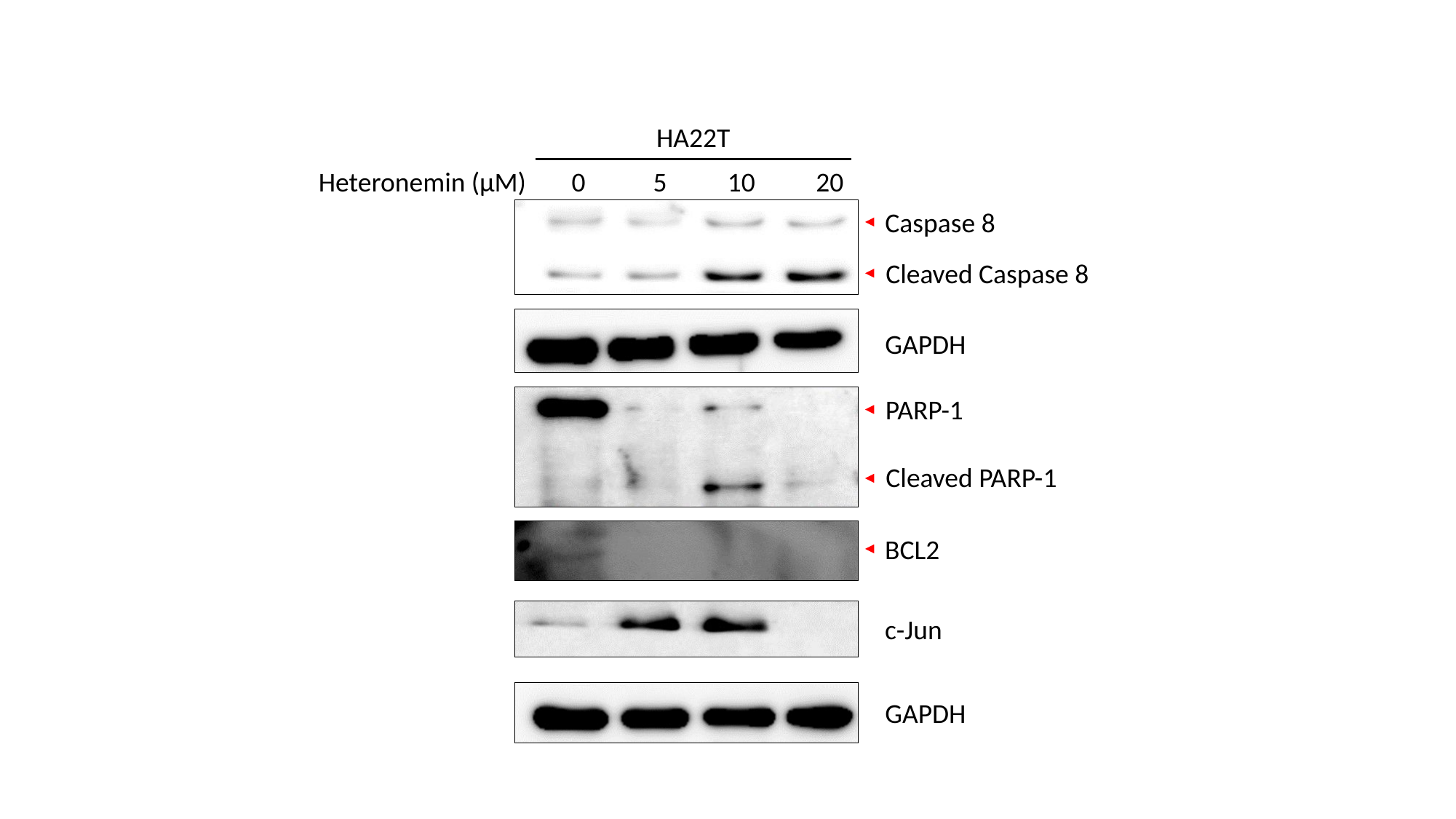

HA22T
Heteronemin (μM)
0
5
10
20
Caspase 8
Cleaved Caspase 8
GAPDH
PARP-1
Cleaved PARP-1
BCL2
c-Jun
GAPDH

Supplement: Supplementary Materials — Supplementary Figure 1: The expression of apoptotic- and MAPK-associated proteins. HA22T cells were treated with indicated concentrations of heteronemin for 14 hours respectively, and the changes of protein expression was determined by Western blot assay. [file 7689045.f1.pptx]
